# Developmental biologists' choice of subjects approximates to a power law, with no evidence for the existence of a special group of 'model organisms'

**DOI:** 10.1186/1471-213X-7-40

**Published:** 2007-05-01

**Authors:** Jamie A Davies

**Affiliations:** 1 University of Edinburgh Centre for Integrative Physiology, Edinburgh, UK

## Abstract

**Background:**

This report describes an unexpected aspect of the structure and development of developmental biology research, rather than the development of a specific embryo. Descriptions of modern developmental biology emphasize investigators' concentration on a small number of 'model' organisms and it is assumed that a clear division exists between the attention paid to these 'model' organisms and that paid to other species. This report describes a quantitative analysis of the organisms that were the subjects of studies reported in developmental biology journals published in the years 1965, 1975, 1985, 1995 and 2005, chosen to represent five decades of modern developmental biology.

**Results:**

The results demonstrate that the distribution of attention paid to different organisms has a smooth distribution that approximates to a scale-free power law, in which there is no clear discontinuity that divides organisms into 'models' and the rest. This is true for both individual years and for the aggregate of all years' data. In other systems (eg connections in the World Wide Web), such power-law distributions arise from mechanisms of preferential attachment ('the rich get richer'). Detailed analysis of the progress of different organisms over the years under study shows that, while preferential attachment may be part of the mechanism that generates the power law distribution, it is insufficient to explain it.

**Conclusion:**

The smoothness of the distribution suggests that there is no empirical basis for dividing species under study into 'model' organisms and 'the rest', and that the widely-held view about organism choice in developmental biology is distorted.

## Background

This brief report is about the research discipline of developmental biology, rather than being about the development of any specific organism. It applies a scientific method of analysis to resolve two incompatible but widely held assumptions about the structure and development of the field, specifically the way that attention is focussed on different organisms. The results shed new light on how developmental biologists collectively organize their research.

Both assumptions concern developmental biologists' choice of experimental subject, a key aspect of the structure of any science. It is generally acknowledged that most developmental biology research is performed on a small number of organisms that are genetically tractable, easily manipulated, or relevant to human biomedicine. The organisms are often referred to as 'model organisms', with the term 'model' being used to signify universality of developmental mechanisms within a broad taxonomic group [1], even though this has been argued to be a misuse of the word 'model' [2]. Examples include the mouse *Mus musculis*, the fruit fly *Drosophila melanogaster *and the worm *Caenorhabditis elegans*. The extent to which it is sensible to concentrate so much research effort on a limited number of species continues to be a matter for debate [3-7], but there is a strong consensus that these 'model' organisms are easily identifiable as such and that a clear division exists between the attention paid to them and to any other species [1,8-12]. One recent commentary in *Science*, for example, likened the hegemony of *"this handful of organisms to the Security Council of the United Nations because, among the world's multitude of organisms, they garner most of the attention of researchers and dictate the distribution of most of the biomedical research funds that are not targeted to specific diseases" *[13].

Developmental biologists choose their organisms according to a number of criteria that include rapid development, uniformity of between individuals, ease of surgical manipulation, ease of genetic manipulation, suitability for teaching and, for clinically-orientated researchers, potential relevance to human congenital disease[12,14,15]. The choice of organisms has also been historically contingent on such things as forceful personalities, imperial exploration, the chance appearance of a mutation, and good culture methods having been established for purposes irrelevant to developmental biology (eg *Xenopus *in pregnancy testing) [11,14]. While popular models certainly have advantages in terms of ease of husbandry or breeding, many other possible species and genera might have been used instead. The reason generally given for some organisms having become established as 'models' is that the investment already made in understanding them made them more attractive as experimental subjects than related creatures about which nothing was known; in genetics particularly, there is also the effect that probability of particular mutations being discovered scales with the total number of animals that are being bred worldwide [14]. The years of investment in, for example, *Drosophila *genetics, *Caenorhabditis *lineage studies and mouse reproductive technology has made these animals much easier to use for the next step of research than their close relatives would be, and each new step of research has made them yet more attractive. The presence of experienced PhD supervisors who already specialize in one organism may also steer the careers of their students in that direction. The popularity of the organisms might therefore have grown according to the principle of "the rich get richer" (sometimes called the 'Matthew Principle', in a humorous allusion to a biblical quote "*To him who has, more shall be given*": Matt 25: 29).

These two assumptions – (a) that there is a sharp distinction between the attention paid to 'model organisms' and to others, and (b) that creatures become 'model organisms' because attention already paid to them makes them more attractive for future attention – are, however, unlikely to be simultaneously true. Many physical, technological and socio-economic systems feature the Matthew Prinicple, new entities being most likely to attach to the most dominant existing entities in a process usually called 'preferential attachment'. Already highly-linked websites attract more new links than less-linked websites do, airports with many onward connections are most attractive to airlines choosing new routes, and the richest people are able to become richer by investing their existing wealth [16-21]. All of these cases are relatively well-understood and modelled. In none, though, does the process of preferential attachment produce a sharp distinction between entities at the top of the distribution (those most-connected, richest etc) and the rest. Instead, the distribution of connections, wealth *etc*. follows a continuous power law that is scale-free, its shape being identical across the distribution [16-21].

To resolve this apparent contradiction, I have examined the literature of developmental biology to discover how attention is really divided between different species. The data show no evidence of a distinction between 'model' and other organisms but instead approximate to a continuous power law. This argues against there being any empirically-defensible division between 'model organisms' and others. Detailed examination of the interest in different organisms at different times shows, however, that the power law does not arise through a simple process of preferential attachment in which interest in any organism at one time can be predicted accurately from interest in it at a past time.

## Results and discussion

The 4615 developmental papers published in the five years combined (1965, 1975, 1985, 1995, 2005) focused on a total of 287 genera that ranged from Acetabularia to Zea. Two genera, *Mus *and *Drosophila*, accounted for two fifths of papers published, and these two plus *Gallus*, *Xenopus*, *Brachydanio*, *Rattus *and *Caenorhabditis *together accounted for more than two thirds of all papers. This statistic probably accounts for the widespread, subjective perception that there is a special group of model organisms. There was, on the other hand, a large number of genera such as *Lumbriculus*, each of which individually received little attention. The rarer species usually appeared in their own right and not just as comparison organisms in papers concentrating on another organism. The great majority of papers focused on one organism only; in 1965, for example, only 8 of 157 papers studied more than one organism, and this ratio is typical of all years studied.

**Figure 1:**
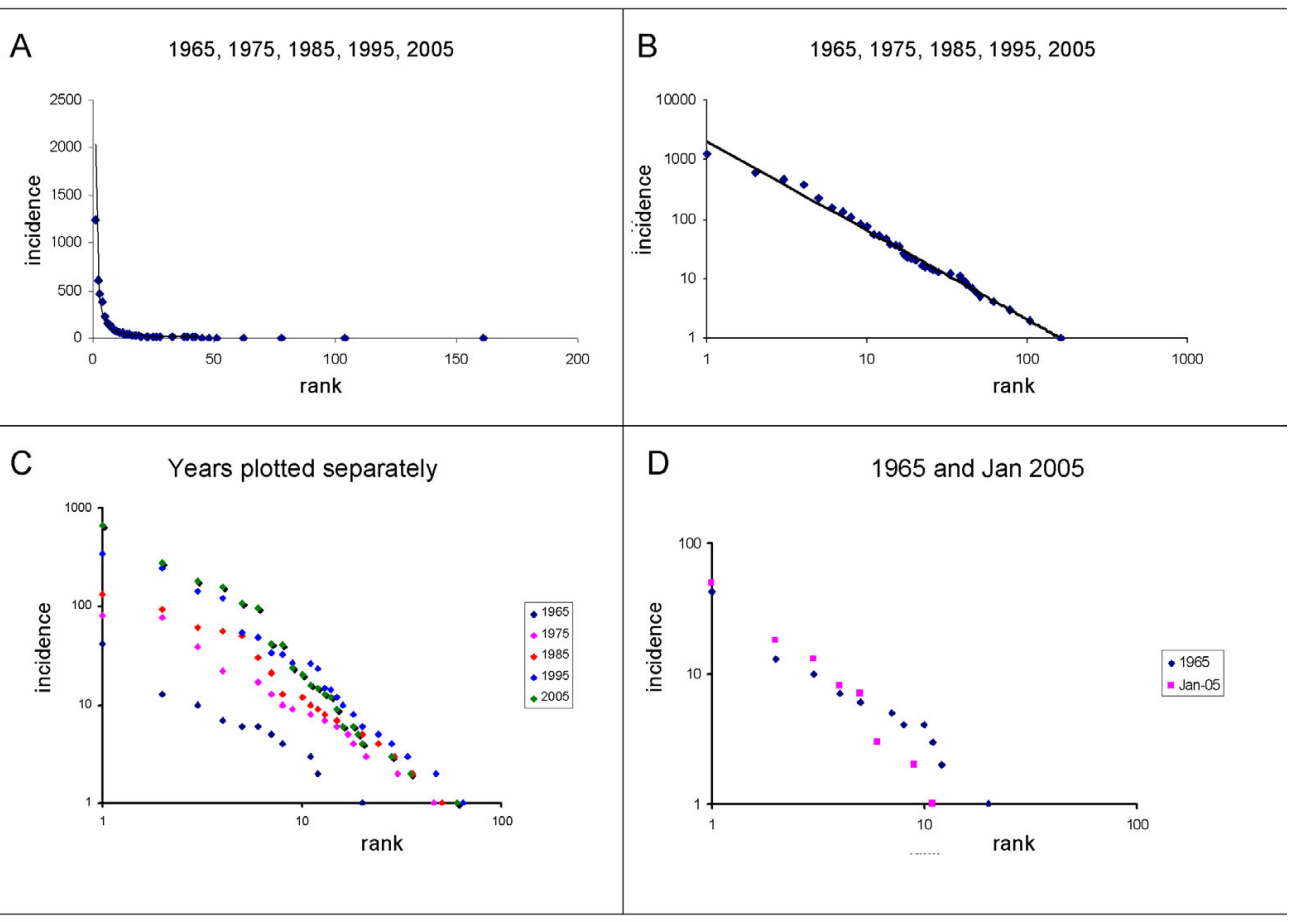
The distribution of attention given to different genera in the developmental literature. 1a shows a linear plot the incidence of a genus being studied in any of the five years under study versus the rank of that incidence (most studied = rank 1, second-most = rank 2 etc). Fig 1b shows a Zipf plot (*i.e*. log-log, incidence versus rank) of the aggregate data from developmental papers in 1965, 1975, 1985, 1995 and 2005. Dots show the data, and the line shows a power-law trend line. 1c shows a similar plot of each year plotted separately; the trends of each set of data each approximate to a power law trend line for that year, the lines of each set of data being approximately parallel. The progressive diagonal shift from the origin reflects the increasing size of the developmental biology literature (157 papers in 1965, 1838 in 2005). This is illustrated by 1d, in which the data for just January 2005 fall on a line about as far from the origin as the data from the whole year of 1965.

A linear plot of the number of papers focusing on an organism, versus the rank of that organism, illustrates the extent to which attention is and has been focused on comparatively few genera (Fig 1a). A Zipf plot (log/log) of the same data, however, shows that the trend of the data approximates to a scale-free smooth power law, *incidence *= *2031·rank*^-1.5 ^(Fig 1b) with no obvious discontinuities. A linear regression analysis of log(incidence) vs log(rank) yields a correlation coefficient to this power law trend of r = 0.999. Analysis of the frequency distibution of the data using the LOTKA program [22], which assesses goodness of fit of data to a power law by a Kolmogorov-Smirnov test designed for the purpose, yielded a maximum deviation of 0.049; the critical value for p = 0.01 is 0.096, so the fit of the data to a power law can be accepted at p < 0.01.

Plots of the individual years (Fig 1c) show a broadly similar pattern for each year, the different displacements of the different years' points from the origin reflecting the different total numbers of papers (as illustrated by Fig 1d). The trends of data for individual years (particularly 1985) are not quite as smooth as those of the grand total, as might be expected with smaller data sets. The small irregularities in these annual curves do not occur in the same places in each year, however, which argues against any steady pattern of deviation from the power law and explains why the grand total (Fig 1a) is smoother. In any case, the fit of the data of each individual year to its own power law is confirmed at p < 0.01 by the Kolmogarov-Smirnov test in the LOTKA program [22]. Inclusion of the evo-devo data for 2005 makes no significant difference to the 2005 power-law curve.

If the widely-held assumption about the existence of 'model organisms' were true, one would not expect a smooth, scale-free distribution. There might be, for example, a relatvely flat line, high on the y axis, for the few 'model organisms' and then a steep drop to a relatively flat line low on the y axis for the rest. In fact, there is no discontinuity but instead a remarkably good approximation to a smooth power law (Fig 1a). There is therefore no empirical justification for dividing the organisms studied by developmental biologists into 'model organisms' and others without invoking some arbitrary and subjective cut-off, such as 'those that account for more than 2/3 of the literature'. That is the first main conclusion of this paper.

**Figure 2:**
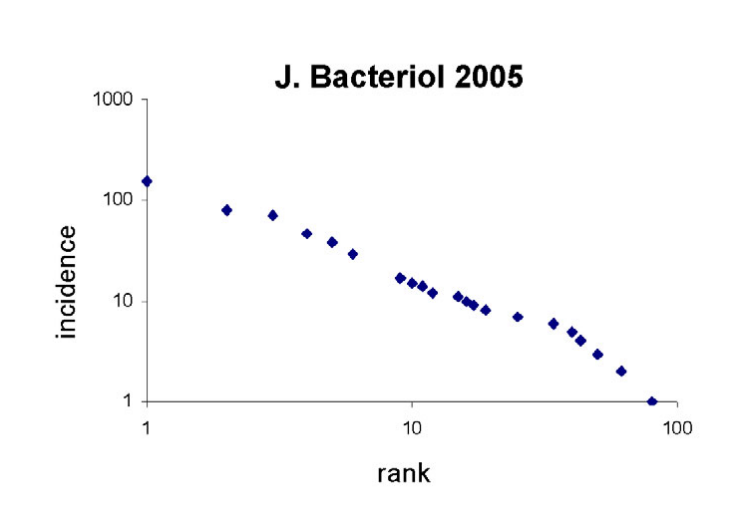
The distribution of attention given to different genera of bacteria, in all papers in *Journal of Bacteriology *published in 2005. Again, the data approximate to a power law, with correlation r = 0.974, showing that this pattern is by no means restricted to developmental biology. Again, some points contain multiple independent entries of the same incidence and therefore rank.

It is interesting to note that such a distribution of attention to different organisms is not restricted to developmental biology. Bacteriology is another subject in which studies are made of a number of different organisms. Fig 2 shows a Zipf plot of the attention given to different microorganisms by bacteriologists publishing in the *Journal of Bacteriology *in 2005. Again, the data approximate to a power law (p < 0.01).

**Figure 3:**
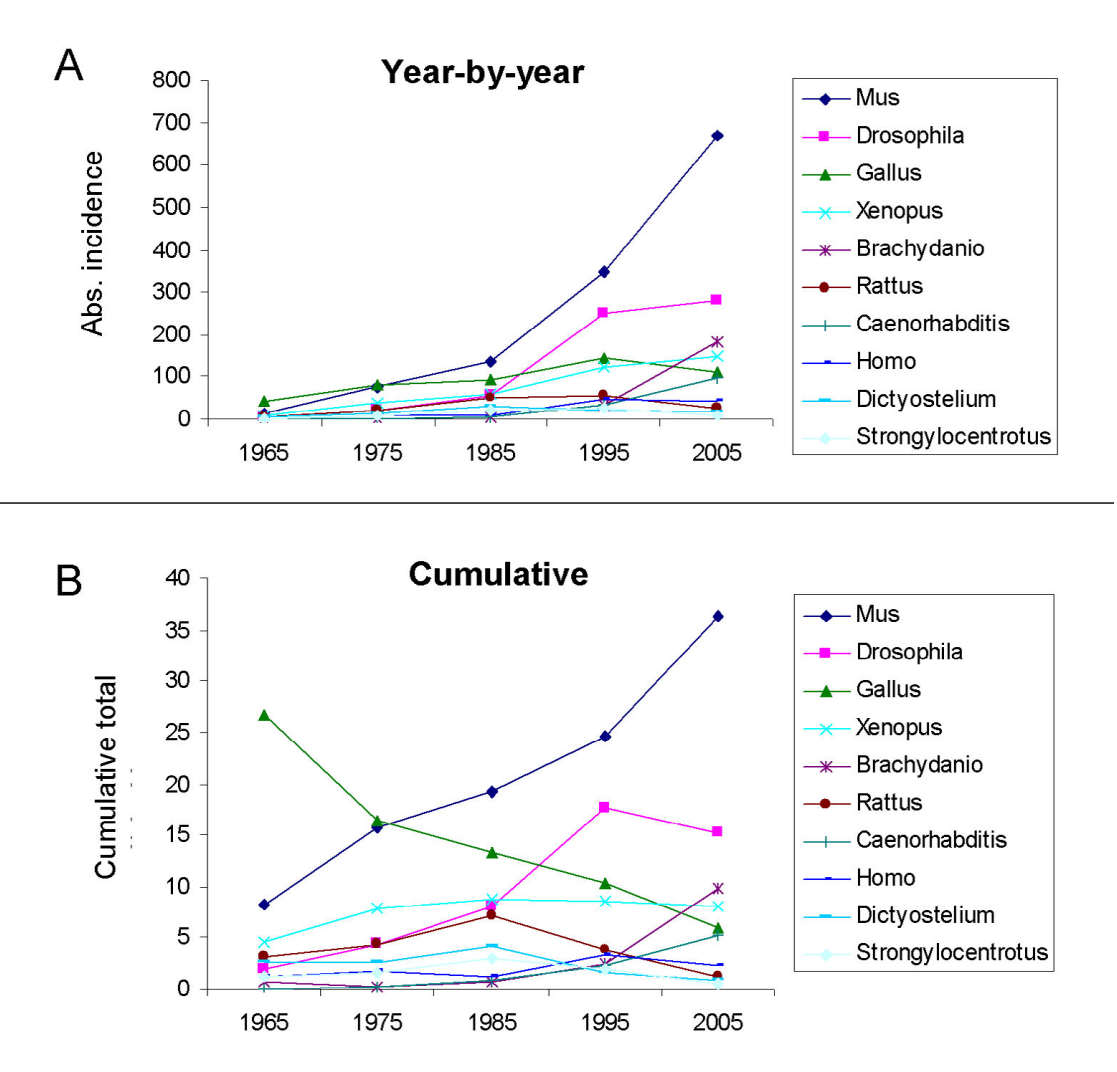
The changing fortunes of the 'top ten' organisms (see table 2) between 1965 and 2005. (a) shows the absolute incidence of each of these organisms appearing in a paper in each of the years under study. (b) shows the cumulative incidence of the data in each of these years. It is clear that the rate of change of attention lavished on any organism in one year cannot be deduced simply from the data of an earlier year. The attention paid to some organisms (eg Mus) increases consistently while that paid to others (eg Gallus and Rattus) rises then declines: significantly, lines cross, which they would not do in a simple mechanism of preferential attachment.

Power law distributions often arise by mechanisms of preferential attachment, as seems to be the case in accumulation of fiscal wealth or the accumulation of connections in a computing or transport network [16-21]. The discovery of a power law in the distribution of researchers' attention to different organisms seems, at first sight, to support a mechanism of preferential attachment in which the more research attention an organism has attracted, the more attractive it becomes to future researchers [19,23,24]. This idea can be tested by study of the changing attention being paid to individual organisms through time; in a mechanism of preferential attachment, attention being paid one year should be predictable from the attention paid in a previous year. To avoid the problem of trying to analyse historical trends of organisms that appeared in papers only once or twice in the five years under study (where no meaningful trend could be visible), the changing fortunes of only the 'top ten' organisms (table 1) were plotted. The changing year-by-year values and cumulative totals are shown in Fig 3. It is clear that some organisms gain attention more quickly than others, and the attention given to others even declines resulting in lines crossing each other. In particular, *Drosophila *and *Brachydanio *show rapid period of expansion that cross the lines of previously-established organisms, possible because of the 'opening up' effect of large-scale gene screens performed on these organisms by one or two specialist labs. In a simple mechanism of preferential attachment, in which the probability of a new paper focusing on a particular organism is proportional to the number of papers that have already focused on that organism, lines should not cross. The power law distribution of attention to different organisms cannot therefore be explained by simple mechanism of preferential attachment. That is the second main conclusion of this paper.

**Table 1:** The 'top ten' organisms from the aggregate of the five years (1965, 1975, 1985, 1995, 2005) under study.

Genus	Incidence	Incidence (%)
*Mus*	1241	26.9
*Drosophila*	609	13.1
*Gallus*	471	10.2
*Xenopus*	377	8.2
*Brachydanio*	221	4.8
*Rattus*	157	3.4
*Caenorhabditis*	136	2.9
*Homo*	109	2.4
*Dictyostelium*	85	1.8
*Strongylocentrotus*	76	1.6

It may not, on reflection, be that surprising that the attention focused on different organisms cannot be explained entirely by a simple process of preferential attachment. In 1929, well before the alleged era of 'model organisms' [1], August Krogh suggested that, for most biological problems, there will be an organism that is ideal for study of that problem [25]. This has become known as the "Krogh principle" [26]. If the Krogh principle still holds true, the statistics of organism choice will follow the statistics of problem choice. Research interest in a scientific problem might grow by preferential attachment, but this can last only until the problem has been solved; at that point, attachments to the problem cease and researchers will publish papers about different problems. Some will choose these problems with no organism bias, while others will feel attached by investment in one organism (in fish breeding tanks, for example) to choose another problem for which the same organism is suitable. Overall, there are therefore likely to be several processes of attachment and detachment operating at the same time, each with its own focus and range of time-scales, which can account for interest in some organisms rising while that in others falls.

That smooth power-law distributions can be maintained under circumstances that involve multiple complex processes is known for other systems. In economics, for example, the distribution of wealth among individuals was shown to approximate to a power law in 1896 [17] and in 1999 [27] despite the fact that the wealth-holding individuals will have been completely replaced over that 103-year period and the mechanisms regulating the fortunes even of families will have been very complex through the wars, booms and slumps of the 20^th ^century.

The main finding of this report – that research interest in organisms approximates to a smooth power law and there is no empirical evidence for the existence of a set of 'model organisms' – may not remain true. The sponsors of large-scale genomics and database projects initially targeted whole-genome sequencing to a limited number of organisms (that they call 'model organisms') and support organizations echoed this concentration of resources. The MeSH term policy of the National Library of Medicine (USA) is an example, in which some types of record are given for proteins only if they come from one of 11 listed organisms [28]. If policy were to continue to concentrate genomic and bioinformatics resources on just these organisms, then eventually even the Krogh principle would repeatedly direct researchers to these because these resources will give an informatic advantage to them, even if they are awkward in other ways. If, however, the rate at which genomes can be sequenced continues to increase at its current rate (doubling every year [29]) it may be feasible for the genomes of very large numbers of organisms to be sequenced quickly and easily. Indeed, this is already happening for prokaryotes, for which over 200 genomic sequences are now available. In that case, the smooth power-law will probably remain, and a balanced study of biology will not be eclipsed by over-concentration on just a few creatures.

## Conclusion

Research interest in organisms approximates to a smooth power law and there is no empirical evidence for the existence of a set of 'model organisms'.

## Methods

For the purposes of this study, the research literature of developmental biology was deemed to consist of, and to be limited to, publications in the journals *Cell Differentiation, Development, Developmental Biology, Developmental Cell, Developmental Dynamics, Development, Growth and Differentiation, Differentiation, Embryologia, Genes & Development, Genesis, Journal of Embryology and Experimental Morphology, Mechanisms of Development, Organogenesis *and *Roux' Archive of Developmental Biology *(see table 2). This simple definition was used to avoid the need for subjective judgements about what was or was not a 'developmental' paper. Evo-devo journals were omitted from the main analysis, as it was felt that their concentration on comparative studies might have biased this study in favour of its eventual conclusion. An supplementary analysis for 2005 with the inclusion of journals such as *Evolution and Development *was, however, also performed (such journals did not exist for earlier years). For all of the manuscripts published in these journals in the years 1965, 1975, 1985, 1995 and 2005, the total number of times that each organism was the focus of experimental attention was recorded. Organisms used in a paper merely as part of a method, and not as the focus of research (for example, *E. coli *when used simply as a cloning tool for mammalian genes, or the host animal for a commercial antibody) were ignored. This is because such organisms are not 'chosen' in the same way as the main topic of research, but are rather dictated by safety legislation, the availability of commercial kits etc. Where an organism such as *E. coli *was itself the subject of research, it was recorded. Organisms were recorded to genus level only, because most reports specified the organism only to the level of genus (eg "*Xenopus*"), not species. A few papers, mainly theoretical or biochemical, could not be ascribed to a particular set of organisms at genus level and these were excluded from the study. Each year, these accounted for fewer than 1% of papers examined. Review papers, editorials and corrigenda were ignored.

**Table 2:** Publication histories of the journals used in this study.

Title	1965	1975	1985	1995	2005
*Dev Biol*	Yes	Yes	Yes	Yes	Yes
*JEEM → Development*	Yes^JEEM^	Yes^JEEM^	Yes^JEEM^	Yes^DEV^	Yes^DEV^
*Embryologia → Dev Grth Diff*	Yes^E^	Yes^DGD^	Yes^DGD^	Yes^DGD^	Yes^DGD^
*Roux Archive Dev Biol*	Yes	Yes	Yes	Yes	-
*Cell Differentiation → Mech Devel*	-	Yes^CD^	Yes^CD^	Yes^MoD^	Yes^MoD^
*Differentiation*	-	Yes	Yes	Yes	Yes
→ *Dev Dynam*	-	-	-	Yes	Yes
*Genes & Development*	-	-	-	Yes	Yes
*Dev Cell*	-	-	-	-	Yes
→ *Genesis*	-	-	-	-	Yes
*Organogenesis*	-	-	-	-	Yes

Arrows between journals depict changes of title, the span of each title being indicated by superscripts in the table. An arrow as the left-most character before the mention of a title shows that the journal is a developmentally-specilized relaunch of a previous journal too general to be included in this study (for example, *Developmental Dynamics*, a fully-developmental journal, is a continuation of *American Journal of Anatomy*, which included significant non-developmental content and was therefore exlcuded from this study).

The genera studied in this body of literature were ranked by the numbers of papers in which they were studied (most studied = rank 1). The number-of-times-studied ('incidence') was then plotted against rank in a linear and in a log-log (Zipf) plot [30]. All data appear on the graphs, although many points overlap especially towards the right side of the graphs as organisms with equal incidence share the same rank. Ranking for the Zipf plot and regression analysis of log [papers] vs log [rank] was done using built-in functions of Microsoft Excel. The graphs presented in Figs 1 and 2 were produced by Excel, using all of the relevant data and plotting to logarithmic axes. A frequency distribution of the data was also produced, using the 'data analysis' extension of Excel, and its fit to a power law was analysed using the LOTKA program produced for this precise purpose [22].

## References

[B1] Churchill FB (1997). Life before model systems: general zoology at August Weismann's Institute. American Zoologist.

[B2] Gest H (1995). Arabidopsis to zebrafish: a commentary on 'Rosetta stone' model systems in the biological sciences. Perspectives in Biology and Medicine.

[B3] Fields S, Johnston M (2005). Cell biology. Whither model organism research?. Science.

[B4] Barr MM (2003). Super models. Physiol Genomics.

[B5] Poccia D (2006). Editorial: Beyond the model organism. J Exp Zoolog A Comp Exp Biol.

[B6] Callery EM (2006). There's more than one frog in the pond: a survey of the Amphibia and their contributions to developmental biology. Semin Cell Dev Biol.

[B7] Glick BS (1996). Cell biology: alternatives to baker's yeast. Curr Biol.

[B8] Gilbert SF, Raunio A (1997). Embryology: constructing the organism ppIX-X.

[B9] Slack JMW (2006). Essential developmental biology (2nd edition) p61.

[B10] Wolpert L (1998). Principles of development pp23-59.

[B11] Gurdon JB, Hopwood N (2000). The introduction of Xenopus laevis into developmental biology: of empire, pregnancy testing and ribosomal genes. Int J Dev Biol.

[B12] Bolker JA (1995). Model systems in developmental biology. Bioessays.

[B13] Fields S, Johnston M (2005). Cell biology. Whither model organism research?. Science.

[B14] Kohler RE (1993). Drosophila: a life in the laboratory. Journal of the History of Biology.

[B15] Ankeny RA (2001). Model organisms as models: understanding the 'Lingua Franca' of the human genome project.. Philosophy of science.

[B16] Amaral LA, Scala A, Barthelemy M, Stanley HE (2000). Classes of small-world networks. Proc Natl Acad Sci U S A.

[B17] Pareto V (1897). Cours e'economique politique.

[B18] Gabaix X (1999). Zipf's law and the growth of cities. American Economic Association Papers & Proceedings.

[B19] Barabasi AL, Albert R (1999). Emergence of scaling in random networks. Science.

[B20] Huberman BA, Adamic LA (1999). Internet - Growth dynamics of the World-Wide Web. Nature.

[B21] Buchanan M (2002). Small World.

[B22] Rousseau B, Rousseau R (2000). LOTKA: a program to fit a power law distribution to observed frequency data. Cybermetrics.

[B23] Newman ME (2001). Clustering and preferential attachment in growing networks. Phys Rev E Stat Nonlin Soft Matter Phys.

[B24] Dorogovtsev SN, Mendes JF, Samukhin AN (2000). Structure of growing networks with preferential linking. Phys Rev Lett.

[B25] Krogh A (1929). The Process of Physiology. Am J Physiol.

[B26] Krebs HA (1975). The August Krogh Principle: "For many problems there is an animal on which it can be most conveniently studied". J Exp Zool.

[B27] Nirei M, Souma W (2004). Two factor model of income distribution dynamics. Complexity Digest: SFI Working Papers.

[B28] Medicine NL (2007). http://www.nlm.nih.gov/pubs/techbull/nd02/nd02_2003_medline_data_changes.html.

[B29] Meyer F (2006). Genome Sequencing vs. Moore's Law: Cyber Challenges for the Next Decade. CTWatch Quarterly.

[B30] Zipf GK (1949). Human behaviour and the principle of least effort: an introduction to human ecology.

